# Personalized Therapy and Liquid Biopsy—A Focus on Colorectal Cancer

**DOI:** 10.3390/jpm11070630

**Published:** 2021-07-01

**Authors:** Niki Christou, Léa Veyrune, Sotirios Georgios Popeskou, Muriel Mathonnet

**Affiliations:** 1 Digestive Surgery Department, University Hospital of Limoges, 87000 Limoges, France; mathonnet@unilim.fr; 2 Laboratoire EA3842 CAPTuR «Contrôle de l’Activation Cellulaire, Progression Tumorale et Résistances Thérapeutiques», Faculté de Médecine, 2 Rue du Docteur Marcland, 87025 Limoges, France; 3 Department of Colorectal Surgery, Queen Elisabeth Hospital, Mindelsohn Way, Birmingham B15 2TH, UK; 4 Service de Cytogénétique, Génétique Médicale et Biologie de la Reproduction, Univerity Hospital of Limoges, 87000 Limoges, France; lea.veyrune@gmail.com; 5 Department of Surgery, Regional Hospital of Lugano, 6900 Lugano, Switzerland; salvator10@yahoo.com

**Keywords:** liquid biopsy, personalized treatment, cancers

## Abstract

(1) Background: Resistance mechanisms represent a barrier to anti-cancer therapies. Liquid biopsies would allow obtaining additional information in order to develop targeted therapies to thwart the resistance phenomena but also to follow in time real response to treatment and be able to adapt it the most quickly possible way in case of resistance. (2) Methods: herein we summarize the different liquid biopsies which are currently under research; we then review the literature and focalize on one of their potential roles: the theranostic one and especially in the cases of colorectal cancers. (3) Results: few studies targeting liquid biopsy as a potential tool to adapt cancer treatments are present in the literature and encompass few patients. (4) Conclusions: further research is needed to prove the efficiency of LB. Indeed, it seems a promising tool to guide treatment by targeting actionable mutations with detection of resistant mutations.

## 1. Introduction

In the last decade, Liquid Biopsies (LB) and their different methods have aroused great interest due to their potential possibility to be both sensible and specific markers for the diagnosis, prognosis and monitoring of cancers. Nowadays, only the CellSearch platform is a validated method for the enumeration of circulating tumor cells (CTCs) in metastatic breast, metastatic colon, and metastatic prostate cancers with the approval of the Food and Drug Administration (FDA) as a useful prognostic method [1]. The clinical implementation of LB is not yet widespread [2]. Despite current research to target the best methodology of LB for the purpose of diagnosis, prognosis or monitoring, several studies have focused on the supplemental aim of LB: adapting treatments as best as possible to provide a personalized choice that will help to avoid resistance to the treatments and therefore recurrences. Herein, we describe LB, its methods and its clinical applications with this specific objective: a personalized cancer treatment. Thus, this mini review aims at providing an overview on the use of liquid biopsy in monitoring treatment response and detection of secondary resistance in metastatic colorectal cancer patients.

## 2. Liquid Biopsy (LB) and Targeted Approaches for Analysis 

### 2.1. Types of Biopsies 

LB corresponds to the collection of liquids like blood, urine, cerebrospinal fluid, in which may be found tumor-derived material that is going to be analyzed [3]. In blood, we can detect different tumor products: tumor cells, nucleic acids, proteins, and exosomes. The two products that have aroused a lot of interest in the last 10 years are both circulating tumor cells (CTCs) and circulating tumor DNA (ctDNA) [4]. Circulating tumor RNAs, because of its fragility, remains the domain of very/high specialized centers.

All these products, coming from either the primary tumor, recurrences or metastases, are present within peripheral venous blood and can be studied even when there is no accessible tumor on which to conduct a biopsy. Contrary to tissue biopsies, without any danger for patients, blood tests can be performed at any moment and can be repeated at any time (Table 1).

#### 2.1.1. Circulating Tumor Cells (CTCs)

##### Origin of CTCs

The first description of CTCs was introduced in 1869 by Ashworth T., an Australian pathologist who discovered them in the blood of a deceased patient and called this phenomenon carcinocythemia [5]. The origin of CTCs is linked to a phenomenon called Epithelial to Mesenchymal Transition (EMT).

##### Epithelial to Mesenchymal Transition (EMT)

CTCs therefore represent the cells within the tumor allowing its propagation. To form distant metastases, cells must be able to detach from the tumor and enter the systemic circulation (intravasation) and then spread and form secondary tumors (extravasation) [6]. Metastases, responsible for around 90% of cancer-associated mortality, are therefore linked to a phenomenon of invasion and colonization by the primary tumor. This is in part linked to a possible cell de-differentiation through aberrant activation of an EMT program. The cells therefore acquire the “stem cell” phenotype (and are cancer initiating cells, “CICs”) allowing them mobility, the basis of the metastatic process [7]. This is largely related to multiple transcription factors such as SNAIL, ZEB and TWIST. SNAIL, for example, is a zinc finger transcription factor and causes suppression of the expression of E-cadherin in different types of cancers such as breast, bladder, stomach and colorectal cancers. Cells must therefore acquire the characteristics of increased motility and invasion in order to propagate, and therefore of multipotency, like “stem” cells. EMT thus allows fixed and polarized epithelial cells, which are linked laterally through several types of junctions and normally interact with the basement membrane, to undergo multiple biochemical changes: loss of cell adhesion, loss of “apex-base polarity”, cytoskeletal remodeling, acquisition of mesenchymal characteristics such as enhancement of migratory capacity, invasiveness, high resistance to apoptosis and increased production of extracellular matrix components. 

##### EMT, Stem Cells and Pathways

This EMT process, which consists of the transformation of epithelial cells into mesenchymal cells with increased migration and invasion properties, appears to be regulated by “stem cells” signaling pathways [8]. Major signaling pathways including TGFβ, Wnt are thus involved in EMT and play a key role in tumor progression [9]. The TGFβ-Smad signaling pathway represents an essential and heterogeneous driver in EMT and colorectal carcinogenesis [10]. TGFβ ligands induce dimerization of TRI and TRII receptors within the membrane, which leads to phosphorylation of Smad proteins [11]. Activated, Smad2 and Smad3 move to the nucleus with Smad4 to serve as a transcriptional regulator [12]. Alterations in TGF receptors and Smad signaling have been detected in advanced adenomas and affect 40–50% of all CRCs [12]. Loss of Smad4 occurs in 30% of metastatic CRCs and is significantly correlated with loss of E-cadherin and increased levels of catenin [13]. The WNT signaling pathway also contributes to the progression of CRC and the regulation of EMT. Its aberrant activation is characteristic of CRCs with mutations in APC or β -catenin. In fact, there is an increase in nuclear β-catenin in tumor cells of the mesenchymal phenotype undergoing active EMT at the tumor front accompanied by changes in the expression of E-cadherin [7]. Various studies have also been able to demonstrate the role of the RAS–ERK 1-ERK 2 signaling pathway in the EMT of CRCs. For example, the *BRAF* and *RAS* oncogenes regulate RhoGTPases to mediate cell migration and invasion, alone, or in relation to the TGF beta pathway [14]. Activation of the PI3K-AKT pathway through *PI3CA* mutations or loss of *PTEN* is associated with colorectal tumor progression [15]. However, the cell under the mesenchymal phenotype will not be able to proliferate and metastasize, which is why it finds itself forced to undergo a new transition, but this time of the mesenchymal-epithelial type (MET). 

##### Mesenchymal-Epithelial Type (MET)

This phenomenon is so important that pharmaceutical companies are taking a very close keen interest in molecules that prevent this mesenchymal transformation into the epithelial phenotype. In fact, we notice that between 30 and 40% of patients, when they present themselves, are already metastatic. In addition, if there is no metastasis, there are at least circulating tumor cells. As long as these cells do not have to undergo the process of mesenchymal transformation to the epithelial phenotype, the formation of metastases can be avoided. For this reason, many drugs currently being tested to stop this transformation are being studied, suggesting the “reversibility of the EMT system”: The overall EMT process thus allows CICs to produce more differentiated progenitors but also to convert non-cancer initiating cells to CICs. Therefore, all of these factors contribute to variations in time in the CIC population, and as a consequence this can lead to resistance to therapy. 

##### Characteristics of CTCs, Methods of Detection and Challenges

CTCs are present in very small quantities in the blood: 1 CTC for 10^7^ leukocytes/mL of blood. They are very rare, “embedded” in other blood nucleated cellular elements, white blood cells [16]. Therefore, due to this rareness, their detection always goes through an enrichment and a selection phase. 

The methods used to lead to enrichment and detection are based on the immunological properties or biophysical properties of these cells. Each method has its own advantages and drawbacks. 

Based on their physical properties, we can use:

- Concentration gradient, Oncoquick**^®^,** because CTCs have a higher density than other cellular types [17]. While it was one of the first techniques that was developed, it is not seen as the most efficient. For example, in comparison with CellSearch^®^ (Menarini Silicon Biosystems), in a cohort of 61 patients with cancers, only 23% of patients were found with at least 1 CTC with this technique while Cellsearch^®^ (Menarini Silicon Biosystems), detected at least one CTC for more than 50% of patients [18].
- Filtration technique, Iset**^®^** [19], Rarecells Diagnostics SAS, since CTCs are larger than the other elements of the blood such as white cells. The main advantage of this technique relies on its independence from the presence of specific tumor cell markers. However, a drawback exists: some tumor cells are small and pass through filters [20].
- Electrical features specific to CTCs lead to their discrimination from other cells using dielectrophoresis [21]. As it is a label-free method, it sorts cells independently from cell membrane markers such as EpCAM and is very specific [22].

Thanks to their biological properties, the concept of immunoaffinity can be used. On the one hand, positive selection enrichment methods (CellSearch^®^ [23], CellCollector^TM^, Ephesia) are based on immunoseparation using magnetic beads conjugated to an antibody directed against specific antigens of tumor cells, called “tumor-associated cell surface antigens”, generally EpCAM and cytokeratins (CK8, CK18, and CK19). All the techniques mentioned above target the specific antigen of EpCAM (epithelial cell adhesion molecule), a specific marker of cancer cells. However, by using EpCAM, CTCs that have undergone epithelial-mesenchymal transition (TEM) are not detected. Among all these methods, the CellSearch^®^ system (Menarini Silicon Biosystems) is considered the gold standard for the Food and Drug Administration (FDA) thanks to its characteristics of robustness and reproducibility. On the other hand, negative immunoselection methods (RosetteSep^TM^) [24] are mostly based on a system of depletion of non-tumor cells, which carry specific antigens (such as specific markers for leukocytes). Thus, its main limitation results in lower purity. Moreover, different studies have recently demonstrated that CTCs may be coupled with neutrophils [25] or may fuse with macrophages to form “tumormacrophages” [26]. Therefore, they may be removed from the final sample of this negative enrichment method and consequently missed.

As CTCs present in the bloodstream encompass the heterogeneity of the tumor, in the function of the technology used to detect them, different phenotypes of CTCs can be sorted. 

The prevailing advantage of CTC detection is to provide cells with their integrity, to obtain nucleic acids (DNA and RNA) of good quality and a material analyzable protein. The collection of CTCs allows all techniques adapted to cytology: immunocytology, fluorescence in situ hybridization studies (FISH), molecular biology techniques (DNA sequencing, PCR, RT-PCR, multiplex RNA) and live CTC cultures for pharmacodynamic testing. Thereby, by preserving the molecular identity of the main tumor, they allow a range of analyses including DNA, RNA, and protein levels, as well as functional ones. Moreover, they can surrogate the current methods of follow-up of CRC (with images) leading to earlier diagnosis of recurrences and reduce costs [27].

The main disadvantages linked to the detection of CTCs are the risk of false-negative and false-positive results. Indeed, due to heterogeneity, it is possible that sub-populations cannot be screened or are incorrectly screened. Moreover, CTCs are in low abundance and frail which does not help with their detection. 

#### 2.1.2. Circulating Tumor DNA (ctDNA)

The story of circulating tumor DNA (ctDNA) is also an old one. Circulating free DNA (cfDNA) was first discovered by Mandel in 1948 in the blood of healthy patients [28]. It is a broader term that describes DNA that is freely circulating in the bloodstream, but is not necessarily of tumor origin, in contrast to ctDNA which is considered tumor-derived, fragmented DNA in the bloodstream without being associated with cells. The origin of ctDNA remains unknown and many hypotheses have been put forward. Fragments of these nucleic acids can come from either primary tumor, metastases and/or recurrences present within the plasma and can be derived from different mechanisms:

- Either necrosis or apoptosis of tumor cells in plasma;
- Or excreted from tumor cells within a vesicle called an exosome;
- Or contained within tumor cells.

The detection of ctDNA leads to the highlighting of tumor-derived mutations [29], but can also reveal epigenic aberrations [30] such as structural variance, methylation, and DNA fragment lengths.

There are several important advantages to detecting ctDNA. It is more sensitive to detect disease burden. As with the CTCs, it allows the detection of minimal residual disease after curative treatment. Furthermore, it can predict acquired drug resistance and influence changes in treatment modalities.

However, ctDNA detection presents both false-negative and false-positive results. Indeed, it can be contaminated by normal circulating DNA (false-positive) or not be enough sensitive enough to the type of cancer being detected due to specific mutations (false-negative). In addition, pre-analytical conditions are insufficiently standardized for correctly detecting ctDNA: anticoagulant use, duration, freezing and transport. Furthermore, it is worth noticing that functional assays cannot be performed. 

Through these different methodological approaches to detect these two products, specific molecular information can be provided. Thus, molecular profiling of LB markers may participate in an adaptation of anticancer agents and biological therapies to tailor treatment as specifically as possible to avoid treatment resistance and cancer progression.

### 2.2. Role of Liquid Biopsy in Adapted Cancer Treatment: Targeted Approaches for Analysis

Beyond the interest of both the quantification of CTCs and ctDNA which have been correlated to the aggressivity or the recurrence of the disease thanks to several clinical studies [31], it seems of high importance to use these tools in a qualitative and targeted way. Indeed, a major interest is to determine genotypic and phenotypic heterogeneity of CTCs and ctDNA through molecular and cellular analysis to follow their dynamic changes during cancer management follow-up (with treatment or without) [32].

#### 2.2.1. Targeted Approaches with CTCs

Different methods are able to increase CTC yield, thus facilitating in vitro drug screens on CTCs leading to treatment adaptation. Over the last decade, microfluidic systems such as Parsortix [33], CTC-iChip [34] and the Herringbone chip [35] have been a remarkable tool to enhance CTCs isolation yield from patients samples [36]. By this enrichment of viable CTCs, genomic analysis with drug testing can be performed effectively.

Recently, some teams have tested the possibility of enhancing CTCs yield by combining leukapheresis with ^LP^CTC-iChi [37].

On the other hand, nanomaterials, thanks to their specific ligand binding with CTCs, can recognize CTCs leading to their isolation, detection, characterization, and even to inducing their destruction via their own functional properties. For instance, since the 1960s, liposomes have been the most commonly investigated nanocarrier/drug delivery system to conduct destruction of CTCs. The preferred therapeutic that they carry is tumor necrosis factor (TNF) apoptosis-inducing ligand (TRAIL). In a recent study, the authors conjugated TRAIL on the surface of nanoscale liposomes along with the adhesion receptor E-selectin (ES) which is able to recognize and bind to most of the leukocytes [38]. Then, selectins facilitate deep adhesion to selectin ligands on tumor cells and leukocytes in blood. This process leads to the promotion of TRAIL ligands getting closer to death receptors on the cancer cell surface. The signal for cell apoptosis is consequently initiated. All in all, these nanocarriers liposomes, by targeting leukocytes, enable them to present TRAIL on their surface aiming at killing CTCs. 

#### 2.2.2. Targeted Approaches with ctDNA

In a targeted approach, the sequencing of DNA allows us to outline different specific mutations. It can inform treatment in such situations where mutations are the potential target of different therapeutic agents, contrary to other techniques (NGS-based panels) where many candidates have been interrogated such as am-Seq (Tagged AMplicon deep sequencing), Safe-Seq (safe sequencing system) or CAPP-Seq (Cancer Personalized Profiling by deep sequencing) [39].

Different targeted methods are available. However, two new technologies have improved the sensitivity detection of ctDNA: Droplet Digital PCR (ddPCR) and Beads, Emulsification, Amplification, and Magnetics (BEAMing). These two methods are high sensitive, fast and relatively inexpensive [40].

##### Droplet Digital PCR (ddPCR) 

Derived from the digital PCR, ddPCR utilizes a droplet generator to partition DNA into droplets using an oil/water emulsion. These droplets then have individual polymerase chain reactions. Its sensitivity can vary depending on the percentage of DNA analyzed, but it is around 1 in 10,000 [41]. 

##### Beads, Emulsification, Amplification, and Magnetics (BEAMing) 

Mutations in ctDNA are identified by using flow cytometry. Sensitivity varies between 1.6 in 10,000 and 4.3 in 100,000 [41]. 

## 3. Clinical Applications in Colorectal Cancer: Overview of the Current Literature

Colorectal cancer (CRC) is the third most common cancer worldwide. In France, in 2018, more than 43,300 cases have been diagnosed and around 17,000 deaths were related to it (French National Cancer Institute [INCa], July 2019). Mean age at diagnosis is 71 years. With an overall 5-year survival of 56%, it is the second leading cause of cancer death [42]. It is a cancer classified by INCa with an intermediate prognosis. The specific 5-year survival varies according to the histological stage of the disease at the time of diagnosis, going from 90.8% for the local stages Tis or T1, to 69.5% when there is a loco-regional invasion (stages II or III), and 11.3% for metastatic stages or stage IV. Both sexes combined, it ranks second among cancer deaths, despite the notable progress made over recent decades, when the CRC death rate fell by 25% between the periods 1984–1988 and 2004–2008 [43]. Despite this progress, which has mainly focused on improving perioperative care and the discovery of new systemic therapies, the persistence of a residual disease or the occurrence of a recurrence after a treatment considered as curative can explain the specific survival rate at 3 and 5 years of 79% and 56%, respectively. A figure of 29% of patients who have undergone curative resection develop a recurrence.

Thus, the current burden of cancer treatment lies in the presence of minimal residual disease (MRD). Indeed, in the bloodstream, we find the accumulation of different phenotypic profiles: those of the primary tumor, and those of metastases, which are completely distinct from the initial specimen but also from themselves over time. Heterogeneity is thus prevalent. For instance, in the study of Xu et al., which included 566 metastatic CRC patients, in 5% of cases, *KRAS* in ctDNA from plasma was mutated whereas it was wild-type in the tissue [44].

Until now, both reducing the risk of recurrences and improving overall survival have been linked to the addition of chemotherapeutic agents aimed at the functions of pathological markers such as lymph node status and size of the tumor. Furthermore, the choice of these agents is made according to the mutational status of distinct genes [45,46]. More precisely, it is established that *KRAS NRAS* mutations are negative predictors of response to anti-EGFR treatment for metastatic CRC patients [47], as well as HER2 positivity [48]. On the other hand, antibody anti-HER2 treatment represents a positive therapeutic option for patients who are HER2 positive [49]. The *BRAF* V600E mutation is another marker of predictive negative response to conventional chemotherapies [50]. Microsatellite instability (MSI) is linked to positive response to immune checkpoint inhibitors [51]. 

Thus, it seems of high importance to follow and quantify over time the modifications of biomarkers in LB such as ctDNA and CTCs to detect resistance, monitor disease progression, and adapt treatments according to each specific tumor profile.

Herein, we review the recent studies that have been conducted over the last 5 years, focusing on the use of both CTCs and ctDNA for detecting resistance to treatments and/or disease progression and triggering personalization of treatments.

By using the terms “ctDNA” “CTCs” “resistance” “disease progression”, and “personalization of colorectal cancer treatment” we conducted a literature search within PUBMED EMBASE and ClinicalTrials.gov including all the articles such as randomized controlled trials and clinical trials in English, focusing on colorectal cancer and LB as potential tools to adapt treatments. Twenty-five articles were found, but after careful reading, only 11 articles presented the selected inclusion criteria (Table 2). They corresponded to four clinical trials: the Prospect phase-2 trial: NCT02994888 [52], a Phase 2 Single-Arm Clinical Trial: CRICKET: NCT02296203 [53], a Prospective Ancillary Study to the Unicancer Prodige-14 Trial: NCT01442935 [54] and a phase II Danish multicenter trial (VEK nr. H-KA-20060094) [55]. All these trials encompassed patients with metastatic colorectal cancers (mCRC) and the methodology only focused on ctDNA analysis except for the ancillary study where CTCs were also analyzed.

Consequently, liquid biopsy demonstrates anticipation of tumor progression, informing physicians about the timing to modify clinical treatments and change treatment strategies.

The Danish clinical trial of Spindler et al. [55], by assessing ctDNA, has contributed to outlining the importance of early prediction to treatment response by ctDNA contrary to information brought by tumor sample analysis. Similarly, the ancillary study of prodige 14 has demonstrated this anticipated response to treatment before potential surgery, that is to say with a curative intent and not only at a palliative stage such as the previous clinical trial.

Furthermore, other studies have shown the possibility of molecular modifications during neoadjuvant chemotherapy. Firstly, Cremolini et al. [53], analyzed *RAS* mutations before rechallenge by antiEGFR treatment (cetuximab) for initially *RAS* and *BRAF* wild-type mCRC patients. They showed that patients with ctDNA *RAS* non mutated before rechallenge had a partial response with longer progression-free survival contrary to those with ctDNA *RAS* mutated (median PFS of 4.0 vs. 1.9 months [*p* = 0.03]). Thus, screening of EGFR signaling pathway by LB may contribute to the best adaptation of selection of patients who are best able to benefit from a cetuximab rechallenge. To confirm all these results with robustness, a new trial is upcoming: the CHRONOS (Phase II Trial: Rechallenge with Panitumumab Driven by *RAS* Clonal-Mediated Dynamic of Resistance) study (NCT03227926), in which patients eligible for cetuximab rechallenge are eligible “only if a decrease of at least 50% in the fractional abundance of *RAS* mutations in ctDNA is evident at the time of rechallenge when compared with the time of progression to the first-line anti-EGFR–containing therapy”. Secondly, the recent study of Khan et al., in 2018, [52], analyzed *RAS* mutations in cfDNA of wild-type (WT) mCRC patients treated with a single-agent anti-EGFR monoclonal antibody. They highlighted the presence of subclonal mutations in the *RAS* signaling pathway in cfDNA according to time and space, leading to the prediction of the response to anti-EGFR treatment. 

All in all, these findings suggest the idea that ctDNA analysis is of great interest for monitoring clonal evolution and guiding therapeutic decisions. 

In comparison, few clinical trials (CT) or randomized control trials (RCT) focusing on CTCs and the surveillance of disease progression and/or adaptation of treatments have been found in the literature search. While different retrospective studies have demonstrated that the number of CTCs are linked to the prognosis of any kind of cancer (colon [65], lung [66], breast [67], gastric [68], or urothelial [69] cancers), herein we have emphasized the interest of clinical research studies with well-conducted methodologies (such as CT or RCT). Indeed, these studies have highlighted the interest of looking for targeted expression genes in CTCs. Target gene expression is frequently negative in the primary tumor leading to treatment issues if metastasis is present. For example, estrogen receptors were negative in 40% of breast cancer cases in the primary or metastatic lesion, but positive in detecting CTCs [70]. In the study of Ning et al. in 2018 [63], by measuring the mRNA expression of stem cell (ALDH1) and EMT (PI3Ka, Akt-2, Twist1) markers in CTCs, they showed that Akt-2 expression may predict PFS in mCRC patients receiving different standard and/or experimental treatments. In the MINOAS trial [64] in 2019, where mCRC patients were treated with first-line FOLFIRI/aflibercept, the steady status over time of CEACAM5 mRNA-negative CTCs predicted better OS in comparison to constant detection of positive CTCs (*p* = 0.015). These results have demonstrated that CTCs can be a tool of applied value for prognostic evaluation, but also for effective individual targeted treatments.

## 4. Contribution of the Omics to Personalize the Treatments

In the end, it is the contribution of the omics that allows personalization of the treatments.

Indeed, omics, which encompass various branches of biology such as genomics, transcriptomics, proteomics, metabolomics, glycomics, and lipidomics, lead to improvements in both the quantitative and qualitative characterization of biological molecules with a translation into the structure, function and dynamics of organisms.

Many technological advancements in each branch, as indirectly shown in the paragraphs above, result in the construction of models that can predict the evolution of the disease and allow us to adapt our treatments.

More precisely, we can cite genomics, transcriptomics, proteomics, and metabolomics as areas that have had a great impact. 

Thus, the entire genome can now be quickly analyzed in a cost-effective way thanks to improvements linked to microarrays, RT-PCR, and NGS [71]. In addition, due to its high accuracy, specific and less frequent mutations present in CRC can be highlighted as well as the most frequent ones such as *RAS, RAF, PI3KA*, and *TP53* [72].

Similarly, transcriptomics with microarray, real-time PCR, and RNA-Seq have induced more precision regarding gene expression. In CRC, different splice variants have been demonstrated [73]. Heterogeneity is thus clearly present at the RNA level [74].

Despite the importance of mass spectrometry (MS) in bringing information concerning protein expression [75], such as different structural and quantitative modifications, to the fore in CRC treatment [76], some limitations have recently been pointed out relating to its lack of specificity, which is not as high as that of genomics [77]. Metabolomics lead to enriched proteomics data, which underline the changes at a cellular level generated through both genetic and proteomic modifications. Many studies have recently shown the importance of glycosylation in CRC [78,79] at various steps: diagnosis, prognosis, and therapeutic.

Consequently, thanks to the compilation and integration of all these individual omics data through computational biology tools, personalized management was born. Indeed, these signatures showing a unique individual with specific and heterogeneous features describing his or her cancer allowed the initiation of this management into the clinic.

## 5. Conclusions

Cancers appear to be dynamic and heterogeneous diseases within space and time. Highlighting biomarkers linked to these tumor evolutions seems challenging. However, in recent years molecular biology technologies have greatly improved. The search for prognostic or predictive factors for optimizing and personalizing treatments is now a real oncological aim. The capability of LB to identify in real-time and in a non-invasive way the different targets of cancer initiation and progression enables us to trigger individualized, adapted therapies. The choice between ctDNA and CTCs assays or their combination with different gene panel analysis in future prospective work will need to weigh up the advantages and drawbacks in terms of sensitivity, specificity, ease of availability, cost, and practicalities with standardization for implementation in clinical practice. Further prospective clinical studies are needed to confirm their clinical applications.

## Figures and Tables

**Table 1:** Liquid biopsy: CTCs and ctDNA.

	CTCs	ctDNA
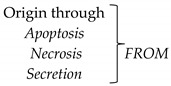	Tumor cells Metastatic cells	Tumor cells Metastatic cells CTCs
Mode of isolation for further analysis	-Direct visualization -Physical separation -Biological separation/immunoaffinity	Nucleic acid samples pre-kit
Analysis capability	-Phenotypic studies -Proteomics -Genomics -Transcriptomics	-Genetics -Epigenetics
Technologies available	-FISH -Flow-cytometry in vitro/in vivo studies -RT-PCR	-NGS -RT -PCR -ddPCR
Pros	-Wide analysis -Functional analysis possible -Molecular analysis at both cellular and sub-cellular levels	-Abundant -Encompasses genetic heterogeneity
Cons	-Rare and fragile -False-negative and false-positive results	-Pre analytical conditions not standardized
A step forward personalized treatments	Biological and immunological test: -drug sensitivity and drug resistance prediction -CTCs clusters-xenograft models	Molecular testing with targetable genomic alterations: gene expression and mutations, genomic signatures

CTCs, Circulating Tumor Cells; ctDNA, Circulating Tumor DNA; FISH, fluorescence in situ hybridization; NGS, next generation sequencing; RT-PCR, Reverse transcription polymerase chain reaction; ddPCR, Droplet Digital polymerase chain reaction.

**Table 2:** Liquid biopsy and personalization of colorectal cancer treatment in Randmomized controlled trials (RCTs).

Clinical Trial	Inclusion Criteria and Type of Intervention	Main Outcome	Methodology for ctDNA Analysis	Results
NCT02994888 [52]	Patients with *RAS* wild-type (WT), refractory metastatic CRC (mCRC)	Mechanisms of resistance/response to anti-EGFR therapies	-ddPCR and ultra-deep next generation sequencing (NGS)	Primary and acquired resistance to anti-EGFR: polyclonal, and observed in both tissue and plasma samples
NCT02296203 [53]	Cetuximab plus irinotecan as third-line treatment for patients with *RAS* and *BRAF* WT mCRC who were initially sensitive to and then resistant to first-line irinotecan- and cetuximab-based therapy	Percentage of patients achieving a decrease ≥ 30% in the sum of the longest diameters of target lesions	-ddPCR and ultra-deep next generation sequencing (NGS)	Rechallenge by cetuximab plus irinotecan is an active option for patients with *RAS* and *BRAF* wild-type metastatic colorectal cancer who have acquired resistance to first-line irinotecan- and cetuximab-based therapy but with *RAS* and *BRAF* wild-type circulating tumor DNA at the time of rechallengeLB leads to track molecular events in (ctDNA) through the different lines of chemotherapy
NCT01442935 [54]	Metastatic disease with synchronous or metachronous (>3 months after diagnosis of the primary tumor) non resectable liver metastasis (LM)	Compare resection rates (R0 or R1) for hepatic metastases	-CTC detected with Cellsearch^®^ *-KRAS* ctDNA (droplet digital polymerase chain reaction (PCR)) levels were assessed at inclusion, after 4 weeks of therapy and before LM surgery.	-CTC detection at 4 weeks (≥1 or ≥3 CTC) was not significantly associated with the eventual R0/R1 resection of LM (*p* = 0.06) -Persistently detectable *KRAS* ctDNA at 4 weeks after neoadjuvant chemotherapy was associated with a lower R0/R1 liver metastasis (LM) resection rate Possible selection of eligible patients to thanks to ctDNA
VEK nr. H-KA-20060094 [55]	Patients resistant to 5-FU, oxaliplatin and irinotecan and treated with 3rd line irinotecan and cetuximab	Clinical value of *KRAS* mutations when detected in plasma compared to tumor in patients from mCRC prior to anti-EGFR therapy	RT-PCR	*KRAS* detection in archival tumor tissue showed no correlation to survival, whereas plasma *KRAS* status remained a strong predictive and prognostic factor in multivariate analysis
NCT02870920. [56]	-Patients with CRC receiving all available standard systemic therapies (fluoropyrimidines, oxaliplatin, irinotecan, and bevacizumab if appropriate; cetuximab or panitumumab if RAS wild-type tumors; regorafenib if available)-Randomized in either Combined Immune Checkpoint Inhibition vs. Best Supportive Care Alone	Overall survival (OS)	cfDNA collected prior to study therapy, at 8 weeks, and at the time of disease progression, with GuardantOMNI next generation sequencing 2.15 Mb, 500-gene panel (Guardant Health, Inc)	Patients with Microsatellite Stable (MSS) status, had significantly improved OS with durvalumab and tremelimumab (*p* = 0.02). Patients who were MSS with plasma Tumor Mutation Burden (TMB) of 28 variants per megabase or more (21% of MSS patients) had the greatest OS benefit (*p* = 0.004).
NCT03010722 [57]	Patients with chemorefractory RAS mutant metastatic CRC received Regorafenib	Overall survival (OS) and Progression Free Survival (PFS)	clonal *RAS* mutations by digital-droplet PCR.	*RAS* mutant clones decay in ctDNA after 8 weeks of treatment was associated with better PFS (*p* = 0.01) and OS (*p* = 0.06) => ctDNA may predict duration of anti-angiogenic response to regorafenib
NCT01704703 [58]	*RAS*-mutated patients with nonresectable metastases from CRC	Overall survival (OS) and Progression Free Survival (PFS)	*RAS* testing in ctDNA using BEAMing before first- and/or second-line treatment	*RAS* mutant allele fraction (MAFs) is an independent prognostic factor in both OS (*p* = 0.006) and first-line PFS (*p* = 0.049).
NCT01001377 [59]	-Patients with chemo-refractory wild-type *KRAS* exon 2 mCRC- randomized 1:1 to receive either panitumumab or cetuximab	Overall survival (OS)	-Plasma samples analyzed at baseline and safety follow-up (SFU) by a next-generation sequencing-based approach for extended *RAS* mutant allele frequency (MAF)-Mutational status of EGFR pathway genes was also examined	Despite observed trend of higher *RAS* MAF correlating with worse outcomes, baseline extended *RAS* mutations did not preclude clinical response to panitumumab. Even though baseline mutations in EGFR pathway genes were associated with shorter OS, the prognostic model analyzing mutations as a continuous variable showed that patients who were mutant for EGFR pathway genes but with a low MAF may still derive clinical benefit from panitumumab.
Appelt et al., *2*020 [60]	Patients with MRI-staged T3-4N0-2M0 rectal cancer treated by neoadjuvant chemoradiotherapy	Overall survival (OS) and the rate of distant metastases were compared between meth-ctDNA (hypermethylation of the neuropeptide Y gene-ctDNA) positive and negative patients	ddPCR	Patients with meth-ctDNA had significantly worse 5-year OS
Janowski et al., 2017 [61]	Patients benefited from resin-based yttrium-90 (90Y) radioembolization for unresectable liver metastasis from CRC	Overall survival (OS) and DNA fragmentation index (FI) quantification	circulating cell-free DNA (ccfDNA) concentration and fragmentation index (FI) were measured using quantitative PCR and atomic-force microscopy (AFM)	In the WT and KRAS mutant patients, DNA FI was reduced after treatment. This reduction was associated with an improved OS (*p* = 0.046). Analysis by AFM of paired pre- and post-treatment samples from KRAS mutant and WT patients revealed significant decrease in fragment size in the WT patients (*p* = 0.013).
NCT01531595 [62]	Metastatic colorectal cancer (mCRC) patients with a known *KRAS* mutation in their primary tumor underwent oncological treatment with bevacizumab in combination with alternating capecitabine and oxaliplatin or irinotecan	Compare multiple methods for measuring *KRAS* mutations in periodically collected liquid biopsies	Plasma ddPCR *KRAS* mutation allele frequency (MAF) *versus* Plasma real-time PCR based molecular testing system (Idylla^TM^ ctKRAS cartridge) *versus*Plasma Next-generation sequencing (NGS) [Ion AmpliSeq Hotspot Panel v2, which surveys the hotspot regions of 50 oncogenes and tumor suppressor genes (Thermo Fisher Scientific, Waltham, MD, USA)]	ddPCR and Idylla^TM^ are equally efficient for the detection of *KRAS* mutations in LB from mCRC patients and that ctDNA may indicate the disappearance of treatment responsive *KRAS* positive mCRC clones, thereby serving as an early sign of disease progression
Ning et al., 2018 [63]	mCRC patients having received standard Food and Drug Administration (FDA)-approved therapies (including fluoropyrimidines, oxaliplatin, irinotecan, bevacizumab, cetuximab, panitumumab, regorafenib), or received experimental agents being tested in three phase I or II clinical trials examining 5-FU plus brivanib (NCT01046864), PRI-724 (NCT01302405) and celecoxib plus EpO906 (NCT00159484).	Measure mRNA expression of EMT (PI3Ka, Akt-2, Twist1) and stem cell (ALDH1) markers in CTCs	-CTCs enrichment: negative immunomagnetic selection using anti- CD45 specific antibodies (Dynabeads M-450 CD45 pan Leukocyte, Invitrogen, Waltham, MA, USA) and then, CD45-negative (CD45−) supernatant s transferred for immune separation and selection using Dynabeads (Dynabeads Epithelial Enrich, #161.02, Invitrogen) with a monoclonal antibody towards human EpCAM,-mRNA expression of EMT (PI3Ka, Akt-2, Twist1) and stem cell (ALDH1) markers was measured	Patients with positive CTC Akt-2 expression had a significantly shorter median PFS in multivariable analyses (HR = 1.70; adjusted *p* = 0.041)
NCT02624726 [64]	Patients treated with FOLFIRI (Folinic acid, fluorouracil and irinotecan)/aflibercept (vascular endothelial growth factor (VEGF) inhibitor)	Objective Response Rate (ORR)	Detection of CEACAM5 mRNA-positive CTCs performed using a real-time PCR assay	At preplanned interim analysis, all patients had discontinued treatment and the ORR was 61.3%, crossing the activity threshold for trial discontinuation.Retaining CTC-negative status predicted better OS compared to continuous detection of CTCs (*p* = 0.015)

## Data Availability

Not applicable.

## Abbreviations

BEAMing	Beads**:** Emulsification, Amplification, and Magnetics
cfDNA	Circulating free DNA
CICs	Cancer initiating cells
CTCs	Circulating tumor cells
CRC	Colorectal cancer
ctDNA	Circulating tumor DNA
EMT	Epithelial to Mesenchymal Transition
EpCAM	Epithelial cell adhesion molecule
ECD	Extracellular domain
FDA	Food and Drug Administration
INCa	French National Cancer Institute
LB	Liquid Biopsy (ies)
MET	Mesenchymal-epithelial type
MRD	Minimal Residual Disease
TNF	Tumor necrosis factor
TRAIL	Apoptosis-inducing ligand

## References

[1] Karachaliou N., Mayo-de-las-Casas C., Molina-Vila M.A., Rosell R. (2015). Real-time liquid biopsies become a reality in cancer treatment. Ann. Transl. Med..

[2] Gingras I., Salgado R., Ignatiadis M. (2015). Liquid biopsy: Will it be the ‘magic tool’ for monitoring response of solid tumors to anticancer therapies?. Curr. Opin. Oncol..

[3] Wu J., Hu S., Zhang L., Xin J., Sun C., Wang L., Ding K., Wang B. (2020). Tumor circulome in the liquid biopsies for cancer diagnosis and prognosis. Theranostics.

[4] Wills B., Gorse E., Lee V. (2018). Role of liquid biopsies in colorectal cancer. Curr. Probl. Cancer.

[5] Ashworth T.R. (1869). A case of cancer in which cells similar to those in the tumours were seen in the blood after death. Aust. Med J..

[6] Mundy G.R. (2002). Metastasis: Metastasis to bone: Causes, consequences and therapeutic opportunities. Nat. Rev. Cancer.

[7] Brabletz T., Hlubek F., Spaderna S., Schmalhofer O., Hiendlmeyer E., Jung A., Kirchner T. (2005). Invasion and metastasis in colorectal cancer: Epithelial-mesenchymal transition, mesenchymal-epithelial transition, stem cells and β-catenin. Cells Tissues Organs..

[8] Visvader J.E., Lindeman G.J. (2008). Cancer stem cells in solid tumours: Accumulating evidence and unresolved questions. Nat. Rev. Cancer.

[9] Polyak K., Weinberg R.A. (2009). Transitions between epithelial and mesenchymal states: Acquisition of malignant and stem cell traits. Nat. Rev. Cancer.

[10] Matsuzaki K., Seki T., Okazaki K. (2006). TGF-β during human colorectal carcinogenesis: The shift from epithelial to mesenchymal signaling. Inflammopharmacology.

[11] Shi Y., Massagué J. (2003). Mechanisms of TGF-β signaling from cell membrane to the nucleus. Cell.

[12] Markowitz S.D., Bertagnolli M.M. (2009). Molecular Basis of Colorectal Cancer. N. Engl. J. Med..

[13] Freeman T.J., Smith J.J., Chen X., Washington M.K., Roland J.T., Means A.L., Steven A.E., Timothy J.Y., Natasha G.D., R. Daniel B. (2012). Smad4-mediated signaling inhibits intestinal neoplasia by inhibiting expression of β-catenin. Gastroenterology.

[14] Makrodouli E., Oikonomou E., Koc M., Andera L., Sasazuki T., Shirasawa S., Pintzas A. (2011). BRAF and RAS oncogenes regulate Rho GTPase pathways to mediate migration and invasion properties in human colon cancer cells: A comparative study. Mol. Cancer.

[15] Roy H.K., Olusola B.F., Clemens D.L., Karolski W.J., Ratashak A., Lynch H.T., Smyrk T.C. (2002). AKT proto-oncogene overexpression is an early event during sporadic colon carcinogenesis. Carcinogenesis.

[16] Ross A.A., Cooper B.W., Lazarus H.M., Mackay W., Moss T.J., Ciobanu N., Tallman M.S., Kennedy M.J., Davidson N.E., Sweet D. (1993). Detection and viability of tumor cells in peripheral blood stem cell collections from breast cancer patients using immunocytochemical and clonogenic assay techniques. Blood.

[17] Müller V., Stahmann N., Riethdorf S., Rau T., Zabel T., Goetz A., Jänicke F., Pantel K. (2005). Circulating tumor cells in breast cancer: Correlation to bone marrow micrometastases, heterogeneous response to systemic therapy and low proliferative activity. Clin. Cancer Res..

[18] Balic M., Dandachi N., Hofmann G., Samonigg H., Loibner H., Obwaller A., van der Kooi A., Tibbe A.G.J., Doyle G.V., Terstappen L.W.M.M. (2005). Bauernhofer, T. Comparison of two methods for enumerating circulating tumor cells in carcinoma patients. Cytom. B Clin. Cytom..

[19] Vona G., Sabile A., Louha M., Sitruk V., Romana S., Schütze K., Capron F., Franco D., Pazzagli M., Vekemans M. (2000). Isolation by Size of Epithelial Tumor Cells: A New Method for the Immunomorphological and Molecular Characterization of Circulating Tumor Cells. Am. J. Pathol..

[20] Farace F., Massard C., Vimond N., Drusch F., Jacques N., Billiot F., Laplanche A., Chauchereau A., Lacroix L., Planchard D. (2011). A direct comparison of CellSearch and ISET for circulating tumour-cell detection in patients with metastatic carcinomas. Br. J. Cancer.

[21] Nguyen N.-V., Jen C.-P. (2018). Impedance detection integrated with dielectrophoresis enrichment platform for lung circulating tumor cells in a microfluidic channel. Biosens Bioelectron..

[22] Le Du F., Fujii T., Kida K., Davis D.W., Park M., Liu D.D., Wu W., Chavez-MacGregor M., Barcenas C.H., Valero V. (2020). EpCAM-independent isolation of circulating tumor cells with epithelial-to-mesenchymal transition and cancer stem cell phenotypes using ApoStream® in patients with breast cancer treated with primary systemic therapy. PLoS ONE.

[23] Hayes D.F., Cristofanilli M., Budd G.T., Ellis M.J., Stopeck A., Miller M.C., Matera J., Allard W.J., Doyle G.V., Terstappen L.W.W.M. (2006). Circulating Tumor Cells at Each Follow-up Time Point during Therapy of Metastatic Breast Cancer Patients Predict Progression-Free and Overall Survival. Clin. Cancer Res..

[24] He W., Kularatne S.A., Kalli K.R., Prendergast F.G., Amato. R.J., Klee G.G., Hartmann L.C., Low P.S. (2008). Quantitation of circulating tumor cells in blood samples from ovarian and prostate cancer patients using tumor-specific fluorescent ligands. Int. J. Cancer.

[25] Szczerba B.M., Castro-Giner F., Vetter M., Krol I., Gkountela S., Landin J., Scheidmann M.C., Donato C., Scherrer R., Singer J. (2019). Neutrophils escort circulating tumour cells to enable cell cycle progression. Nature.

[26] Amantini C., Morelli M.B., Nabissi M., Piva F., Marinelli O., Maggi F., Bianchi F., Bittoni A., Berardi R., Giampieri R. (2019). Expression Profiling of Circulating Tumor Cells in Pancreatic Ductal Adenocarcinoma Patients: Biomarkers Predicting Overall Survival. Front. Oncol..

[27] Labib M., Kelley S.O. (2021). Circulating tumor cell profiling for precision oncology. Mol. Oncol..

[28] Mandel P., Metais P. (1948). [Nuclear Acids in human blood plasma.]. C R Seances Soc Biol Fil..

[29] Heitzer E., Haque I.S., Roberts C.E.S., Speicher M.R. (2019). Current and future perspectives of liquid biopsies in genomics-driven oncology. Nat. Rev. Genet..

[30] Feinberg A.P., Koldobskiy M.A., Göndör A. (2016). Epigenetic modulators, modifiers and mediators in cancer aetiology and progression. Nat. Rev. Genet..

[31] Tan Y., Wu H. (2018). The significant prognostic value of circulating tumor cells in colorectal cancer: A systematic review and meta-analysis. Curr. Probl. Cancer.

[32] Yu M., Bardia A., Wittner B.S., Stott S.L., Smas M.E., Ting D.T., Isakoff S.J., Ciciliano1 J.C., Wells M.N., Shah A.M. (2013). Circulating Breast Tumor Cells Exhibit Dynamic Changes in Epithelial and Mesenchymal Composition. Science.

[33] Miller M.C., Robinson P.S., Wagner C., O’Shannessy D.J. (2018). The Parsortix^TM^ Cell Separation System—A versatile liquid biopsy platform. Cytom. Part A.

[34] Yu M., Bardia A., Aceto N., Bersani F., Madden M.W., Donaldson M.C., Desai R., Zhu H., Comaill V., Zheng Z. (2014). Ex vivo culture of circulating breast tumor cells for individualized testing of drug susceptibility. Science.

[35] Wang Z., Wu W., Wang Z., Tang Y., Deng Y., Xu L., Tian J., Shi Q. (2016). Ex vivo expansion of circulating lung tumor cells based on one-step microfluidics-based immunomagnetic isolation. Analyst.

[36] Cho H., Kim J., Song H., Sohn K.Y., Jeon M., Han K.-H. (2018). Microfluidic technologies for circulating tumor cell isolation. Analyst.

[37] Mishra A., Dubash T.D., Edd J.F., Jewett M.K., Garre S.G., Karabacak N.M., Rabe D.C., Mutlu B.R., Walsh J.R., Kapur R. (2020). Ultrahigh-throughput magnetic sorting of large blood volumes for epitope-agnostic isolation of circulating tumor cells. Proc. Natl. Acad. Sci. USA.

[38] Mitchell M.J., Wayne E., Rana K., Schaffer C.B., King M.R. (2014). TRAIL-coated leukocytes that kill cancer cells in the circulation. Proc. Natl. Acad. Sci. USA.

[39] Palmirotta R., Lovero D., Cafforio P., Felici C., Mannavola F., Pellè E., Quaresmini D., Tucci M., Silvestris F. (2018). Liquid biopsy of cancer: A multimodal diagnostic tool in clinical oncology. Ther. Adv. Med. Oncol..

[40] Elazezy M., Joosse S.A. (2018). Techniques of using circulating tumor DNA as a liquid biopsy component in cancer management. Comput. Struct Biotechnol. J..

[41] Butler T.M., Spellman P.T., Gray J. (2017). Circulating-tumor DNA as an early detection and diagnostic tool. Curr. Opin. Genet. Dev..

[42] Bray F., Ferlay J., Soerjomataram I., Siegel R.L., Torre L.A., Jemal A. (2018). Global cancer statistics 2018: GLOBOCAN estimates of incidence and mortality worldwide for 36 cancers in 185 countries. CA Cancer J. Clin..

[43] (2013). La Situation du Cancer en France en 2012. Collection Etat des Lieux et des Connaissances.

[44] Xu J.-M., Liu X.-J., Ge F.-J., Lin L., Wang Y., Sharma M.R., Xu J.-M., Liu X.-J., Ge F.-J., Lin L. (2014). KRAS mutations in tumor tissue and plasma by different assays predict survival of patients with metastatic colorectal cancer. J. Exp. Clin. Cancer Res..

[45] Yamanaka T., Oki E., Yamazaki K., Yamaguchi K., Muro K., Uetake H., Sato T., Nishina T., Ikeda M., Kato T. (2016). 12-Gene Recurrence Score Assay Stratifies the Recurrence Risk in Stage II/III Colon Cancer With Surgery Alone: The sunrise Study. J. Clin. Oncol. Off. J. Am. Soc. Clin. Oncol..

[46] Taieb J., Jung A., Sartore-Bianchi A., Peeters M., Seligmann J., Zaanan A., Burdon P., Montagut C., Laurent-Puig P. (2019). The Evolving Biomarker Landscape for Treatment Selection in Metastatic Colorectal Cancer. Drugs.

[47] Van Cutsem E., Lenz H.-J., Köhne C.-H., Heinemann V., Tejpar S., Melezínek I., Beier F., Stroh C., Rougier P., van Krieken J.H.J.M. (2015). Fluorouracil, leucovorin, and irinotecan plus cetuximab treatment and RAS mutations in colorectal cancer. J. Clin. Oncol. Off. J. Am. Soc. Clin. Oncol..

[48] Sartore-Bianchi A., Amatu A., Porcu L., Ghezzi S., Lonardi S., Leone F., Bergamo F., Fenocchio E., Martinelli E., Borelli B. (2019). HER2 Positivity Predicts Unresponsiveness to EGFR-Targeted Treatment in Metastatic Colorectal Cancer. Oncologist.

[49] Meric-Bernstam F., Hurwitz H., Raghav K.P.S., McWilliams R.R., Fakih M., VanderWalde A., Swanton C., Kurzrock R., Burris H., Sweeney C. (2019). Pertuzumab plus trastuzumab for HER2-amplified metastatic colorectal cancer (MyPathway): An updated report from a multicentre, open-label, phase 2a, multiple basket study. Lancet Oncol..

[50] French A.J., Sargent D.J., Burgart L.J., Foster N.R., Kabat B.F., Goldberg R., Shepherd L., Windschitl H.E., Thibodeau S.N. (2008). Prognostic Significance of Defective Mismatch Repair and BRAF V600E in Patients with Colon Cancer. Clin. Cancer Res..

[51] Overman M.J., Lonardi S., Wong K.Y.M., Lenz H.-J., Gelsomino F., Aglietta M., Morse M.A., Eric V.C., Ray M., Hill A. (2018). Durable Clinical Benefit With Nivolumab Plus Ipilimumab in DNA Mismatch Repair-Deficient/Microsatellite Instability-High Metastatic Colorectal Cancer. J. Clin. Oncol. Off. J. Am. Soc. Clin. Oncol..

[52] Khan K.H., Cunningham D., Werner B., Vlachogiannis G., Spiteri I., Heide T., Mateos J.F., Vatsiou A., Lampis A., Damavandi M.D. (2018). Longitudinal Liquid Biopsy and Mathematical Modeling of Clonal Evolution Forecast Time to Treatment Failure in the PROSPECT-C Phase II Colorectal Cancer Clinical Trial. Cancer Discov..

[53] Cremolini C., Rossini D., Dell’Aquila E., Lonardi S., Conca E., Del Re M., Busico A., Pietrantonio F., Danesi R., Aprile G. (2019). Rechallenge for Patients with RAS and BRAF Wild-Type Metastatic Colorectal Cancer with Acquired Resistance to First-line Cetuximab and Irinotecan: A Phase 2 Single-Arm Clinical Trial. JAMA Oncol..

[54] Bidard F.-C., Kiavue N., Ychou M., Cabel L., Stern M.-H., Madic J., Saliou A., Rampanou A., Decraene C., Bouché O. (2019). Circulating Tumor Cells and Circulating Tumor DNA Detection in Potentially Resectable Metastatic Colorectal Cancer: A Prospective Ancillary Study to the Unicancer Prodige-14 Trial. Cells.

[55] Spindler K.-L.G., Pallisgaard N., Appelt A.L., Andersen R.F., Schou J.V., Nielsen D., Pfeiffer P., Yilmaz M., Johansen J.S., Hoegdall E.V. (2015). Clinical utility of KRAS status in circulating plasma DNA compared to archival tumour tissue from patients with metastatic colorectal cancer treated with anti-epidermal growth factor receptor therapy. Eur. J. Cancer.

[56] Chen E.X., Jonker D.J., Loree J.M., Kennecke H.F., Berry S.R., Couture F., Ahmad C.E., Goffin J.R., Kavan P., Harb M. (2020). Effect of Combined Immune Checkpoint Inhibition vs Best Supportive Care Alone in Patients With Advanced Colorectal Cancer: The Canadian Cancer Trials Group CO.26 Study. JAMA Oncol..

[57] Khan K., Rata M., Cunningham D., Koh D.-M., Tunariu N., Hahne J.C., Hedayat S., Marchetti S., Lampis A., Damavandi M.D. (2018). Functional imaging and circulating biomarkers of response to regorafenib in treatment-refractory metastatic colorectal cancer patients in a prospective phase II study. Gut.

[58] Elez E., Chianese C., Sanz-García E., Martinelli E., Noguerido A., Mancuso F.M., Caratù G., Matito J., Grasselli J., Cardone C. (2019). Impact of circulating tumor DNA mutant allele fraction on prognosis in RAS-mutant metastatic colorectal cancer. Mol. Oncol..

[59] Peeters M., Price T., Boedigheimer M., Kim T.W., Ruff P., Gibbs P., Thomas A., Demonty G., Hool K., Ang A. (2019). Evaluation of Emergent Mutations in Circulating Cell-Free DNA and Clinical Outcomes in Patients with Metastatic Colorectal Cancer Treated with Panitumumab in the ASPECCT Study. Clin. Cancer Res. Off. J. Am. Assoc. Cancer Res..

[60] Appelt A.L., Andersen R.F., Lindebjerg J., Jakobsen A. (2020). Prognostic Value of Serum NPY Hypermethylation in Neoadjuvant Chemoradiotherapy for Rectal Cancer: Secondary Analysis of a Randomized Trial. Am. J. Clin. Oncol..

[61] Janowski E., Timofeeva O., Chasovskikh S., Goldberg M., Kim A., Banovac F., Pang D., Dritschilo A., Unger K. (2017). Yttrium-90 radioembolization for colorectal cancer liver metastases in KRAS wild-type and mutant patients: Clinical and ccfDNA studies. Oncol. Rep..

[62] Holm M., Andersson E., Osterlund E., Ovissi A., Soveri L.-M., Anttonen A.-K., Kytölä S., Aittomäki K., Osterlund P., Ristimäki A. (2020). Detection of KRAS mutations in liquid biopsies from metastatic colorectal cancer patients using droplet digital PCR, Idylla, and next generation sequencing. PLoS ONE.

[63] Ning Y., Zhang W., Hanna D.L., Yang D., Okazaki S., Berger M.D., Miyamoto Y., Suenaga M., Schirripa M., El-Khoueiry A. (2018). Clinical relevance of EMT and stem-like gene expression in circulating tumor cells of metastatic colorectal cancer patients. Pharm. J..

[64] Matikas A., Souglakos J., Katsaounis P., Kotsakis A., Kouroupakis P., Pantazopoulos N., Kentepozidis N., Nikolaidi A., Messaritakis I., Tzovara I. (2019). MINOAS: A Single-arm Translational Phase II Trial of FOLFIRI Plus Aflibercept as First-line Therapy in Unresectable, Metastatic Colorectal Cancer. Target Oncol..

[65] Soster M., Juris R., Bonacchi S., Genovese D., Montalti M., Rampazzo E., Zaccheroni N., Garagnani P., Bussolino F., Prodi L. (2012). Targeted dual-color silica nanoparticles provide univocal identification of micrometastases in preclinical models of colorectal cancer. Int. J. Nanomed..

[66] Krebs M.G., Sloane R., Priest L., Lancashire L., Hou J.-M., Greystoke A., Ward T.H., Ferraldeschi R., Hughes A., Clack G. (2011). Evaluation and prognostic significance of circulating tumor cells in patients with non-small-cell lung cancer. J. Clin. Oncol. Off. J. Am. Soc. Clin. Oncol..

[67] Bölke E., Orth K., Gerber P.A., Lammering G., Mota R., Peiper M., Matuschek C., Budach W., Rusnak E., Shaikh S. (2009). Gene expression of circulating tumour cells and its correlation with tumour stage in breast cancer patients. Eur. J. Med. Res..

[68] Matsusaka S., Chìn K., Ogura M., Suenaga M., Shinozaki E., Mishima Y., Terui Y., Mizunuma N., Hatake K. (2010). Circulating tumor cells as a surrogate marker for determining response to chemotherapy in patients with advanced gastric cancer. Cancer Sci..

[69] Msaouel P., Koutsilieris M. (2011). Diagnostic value of circulating tumor cell detection in bladder and urothelial cancer: Systematic review and meta-analysis. BMC Cancer.

[70] Somlo G., Lau S.K., Frankel P., Hsieh H.B., Liu X., Yang L., Krivacic R., Bruce R.H. (2011). Multiple biomarker expression on circulating tumor cells in comparison to tumor tissues from primary and metastatic sites in patients with locally advanced/inflammatory, and stage IV breast cancer, using a novel detection technology. Breast Cancer Res. Treat..

[71] Gonzalez-Pons M., Cruz-Correa M. (2015). Colorectal Cancer Biomarkers: Where Are We Now?. BioMed Res. Int..

[72] Malapelle U., Pisapia P., Sgariglia R., Vigliar E., Biglietto M., Carlomagno C., Giuffrè G., Bellevicine C., Troncone G. (2016). Less frequently mutated genes in colorectal cancer: Evidences from next-generation sequencing of 653 routine cases. J. Clin. Pathol..

[73] Pira G., Uva P., Scanu A.M., Rocca P.C., Murgia L., Uleri E., Piu C., Porcu A., Carru C., Manca A. (2020). Landscape of transcriptome variations uncovering known and novel driver events in colorectal carcinoma. Sci. Rep..

[74] Sveen A., Kilpinen S., Ruusulehto A., Lothe R.A., Skotheim R.I. (2016). Aberrant RNA splicing in cancer; expression changes and driver mutations of splicing factor genes. Oncogene.

[75] Cifani P., Kentsis A. (2017). Towards comprehensive and quantitative proteomics for diagnosis and therapy of human disease. Proteomics.

[76] Ku X., Xu Y., Cai C., Yang Y., Cui L., Yan W. (2019). In-Depth Characterization of Mass Spectrometry-Based Proteomic Profiles Revealed Novel Signature Proteins Associated with Liver Metastatic Colorectal Cancers. Anal. Cell Pathol..

[77] Álvarez-Chaver P. (2014). Proteomics for discovery of candidate colorectal cancer biomarkers. World J. Gastroenterol..

[78] Madunić K., Zhang T., Mayboroda O.A., Holst S., Stavenhagen K., Jin C., Karlsson N.G., Lageveen-Kammeijer G.S.M., Wuhrer M. (2020). Colorectal cancer cell lines show striking diversity of their O-glycome reflecting the cellular differentiation phenotype. Cell. Mol. Life Sci..

[79] Liu S., Fu Y., Huang Z., Liu Y., Liu B.-F., Cheng L., Liu X. (2020). A comprehensive analysis of subclass-specific IgG glycosylation in colorectal cancer progression by nanoLC-MS/MS. Analyst.

